# Laparoscopic myomectomy – The importance of surgical techniques

**DOI:** 10.3389/fmed.2023.1158264

**Published:** 2023-03-20

**Authors:** Mihai Cristian Dumitrașcu, Cătălin-George Nenciu, Adina-Elena Nenciu, Amalia Călinoiu, Adrian Neacșu, Monica Cîrstoiu, Florica Șandru

**Affiliations:** ^1^Department of Obstetrics and Gynecology, “Carol Davila” University of Medicine and Pharmacy, University Emergency Hospital of Bucharest, Bucharest, Romania; ^2^Department of Obstetrics and Gynecology, University Emergency Hospital of Bucharest, Bucharest, Romania; ^3^Department of Obstetrics and Gynecology, “St. John” Emergency Clinical Hospital of Bucharest, Bucharest, Romania; ^4^Department of Internal Medicine, “Prof. Dr. Agripa Ionescu” Emergency Hospital, Bucharest, Romania; ^5^Department of Dermatology, Carol Davila’ University of Medicine and Pharmacy, Bucharest, Romania; ^6^Department of Dermatology, Elias Emergency University Hospital, Bucharest, Romania

**Keywords:** laparoscopic myomectomy, suture, surgical technique, barbed suture, pregnancy outcome

## Abstract

Laparoscopy is a routine procedure for benign gynecological tumors. Although the laparoscopic approach for myomas is a common procedure, it can be challenging. To improve outcomes, research regarding port access, suture type, morcellation, and complication management remains ongoing. Myomectomy is the main surgical option for patients seeking uterus-sparing procedures to maintain future fertility. The laparoscopic technique is the most important in these cases, given that possible complications can impact fertility and pregnancy outcomes. Herein, we reviewed and collated the available data regarding different suture techniques, including advantages, difficulties, and possible long-term impacts.

## 1. Introduction

In a society where conception has been slowly shifting toward later life, gynecological pathology is more frequently encountered in females who desire to get pregnant. Uterine fibroids can be detected in 70–80% of females during their lifetime (1). In these cases, a decision must be reached regarding the treatment strategy, considering the symptomatology, characteristics of the fibroid nodule, impact on quality of life, and desire for pregnancy.

The treatment options for uterine myomas depend on the severity of the condition, the age and reproductive status of the patient, and the symptoms experienced. Based on FIGO (The International Federation of Gynecology and Obstetrics) staging, the management can start from observation (if the fibroids are small and asymptomatic) and up to surgical procedures like myomectomy or even hysterectomy in specific cases (2). The FIGO classification presents the uterine fibroids according to their localization which is correlated with specific symptoms and has a significant impact on the treatment options.

Treatment strategies for uterine leiomyoma can include medical options (such as oral contraceptives, progesterone, gonadotropin-releasing hormone agonist (GnRHa), selective progesterone receptor modulators, or the combination of relugolix-estradiol-norethisterone), surgical interventions (such as hysterectomy, laparoscopic myomectomy, and hysteroscopic myomectomy), and non-surgical options (uterine artery embolization) (3). Uterine artery embolization is a convenient method to spare the uterus if a patient experiences substantial bleeding (4). In addition to potential complications associated with uterine artery embolization, the main concern is damage to the ovarian vascular supply (5). The symptomatology of uterine leiomyoma is one of the main factors determining the treatment protocol (6). The uterine-sparing surgery approach is addressed, especially in patients who desire reproductive options in the future. Therefore, surgical procedures should be selected considering that the uterus must be able to carry a pregnancy to term without major risk to the mother or fetus. Myomectomy is a medical procedure used to remove fibroid nodules and reconstruct uterine integrity. Moreover, myomectomy has been the elective fertility-sparing procedure for several years, remaining a top-ranking option owing to improved techniques (7–9).

Considering patients who desire to preserve fertility, approaches can differ depending on whether they wish to become pregnant in the immediate future or maintain this option (10). Female subjects who wish to get pregnant within a short duration can be subcategorized into two groups: those who can try getting pregnant despite the leiomyoma and those who need to address the leiomyoma to become pregnant (10). Moreover, some of these patients are diagnosed with uterine fibroid-related infertility. However, the precise mechanism through which leiomyomas alter fertility remains unclear. It has been suggested that a mechanical alteration occurs due to distortion of the uterine cavity, thereby affecting the cervical passage of the sperm and causing a tubal blockage (11). Fibroid nodules have been associated with increased inflammatory processes and vasoactive substances (12).

Even in the absence of infertility as a complication, leiomyomas during pregnancy can contribute to abortion, premature membrane rupture, premature birth, and labor complications (13). Recurrent pregnancy loss associated with leiomyomas can result from surgery prior to further pregnancy (14). Before laparoscopic myomectomy, one or two cycles of ulipristal acetate can be prescribed if the patient exhibits hypermenorrhoea-related anemia (10). The use of GnRHa prior to surgery has been associated with reduced blood loss and decreased uterine adhesion (15), although susceptibility to uterine fibroid recurrence has been documented (16).

Pregnancy outcomes after laparoscopic myomectomy have been discussed in several studies, which have reported improvements in pregnancy rates to various degrees (14). Uterine rupture is the most common pregnancy complication following myomectomy. Since the introduction of laparoscopic myomectomy as a routine procedure for intramural and subserous uterine nodules almost 30 years ago (17), the strength of the uterine scar has presented a major concern. To improve outcomes, an appropriate surgical technique must be used. The surgeon’s experience is valuable, as he can correlate information regarding characteristics of the uterine fibroid and, consequently, adjust the port position, improve the ergonomics of the procedure, and precisely approach enucleation (18).

One, two, or three ports can be used to perform laparoscopic myomectomy. Typically, single-port laparoscopy results in a prolonged operative time (19, 20). Notably, the surgical port can influence the approach to the myoma in terms of traction, manipulation, enucleation, suturing, and morcellation (20–22). After enucleation, extraction of the uterine fibroid can be performed through one port site or colpotomy, followed by morcellation (23), recommending the in-bag strategy for contained morcellation (24).

In exceptional cases, a myomectomy can be performed during pregnancy (25). Considering leiomyoma refractive to conservative management, the laparoscopic approach has been associated with favorable outcomes and reduced complication rates (26).

Enucleation and closing techniques are the main determinants governing the success of laparoscopic myomectomy and subsequent obstetrical outcomes (27).

## 2. Types of sutures

The advantages of the laparoscopic approach for uterine fibroids have been well established. Compared with laparotomy, laparoscopy affords advantages such as a reduced hospitalization period, a minimal decline in hemoglobin levels, and low levels of postoperative pain (28); however, drawbacks such as blood loss and prolonged surgical time need to be addressed (29).

Bleeding control is one of the main challenges during this procedure, especially when it involves larger or more vascular fibroids. Before dissection, there are some techniques that can be used to help reduce blood loss. Placing a tourniquet at the base of the leiomyoma can significantly reduce the blood supply with the most effect on smaller fibroids (30). In case of a larger or more vascular nodule, the intermittent uterine artery clamping can be realized through laparoscopy (31). Intramyometrial injection of vasoconstriction agents (vasopressin, epinephrine) in the myometrium can be used successfully in reducing bleeding during laparoscopy but can lead to severe complications (32, 33). Hemostatic agents, such as fibrin glue, can be used to help control bleeding during laparoscopic myomectomy by promoting clot formation (34). In addition to these techniques, there are several other strategies that can be used to reduce bleeding during laparoscopic myomectomy. For example, ensuring adequate visualization of the surgical field, using a good surgical technique, and being mindful of tissue handling can all help to minimize bleeding. It’s important to note that some bleeding is expected during any surgical procedure, and it’s essential to have skilled and experienced surgical personnel to manage any complications that may arise.

Suturing remains the most important factor, even when employing methods such as ligation of the uterine artery, oxytocin, or vasoconstrictor agents (35, 36). The number of ports and type of suture are closely associated. Given the availability of instruments and suture devices, surgeons attempt to reduce the number of abdominal incisions without increasing operative time. For example, barbed suture devices are typically selected in single-port laparoscopy due to technical difficulties (37).

The first step in optimal suturing is the uterine incision. For posterior and anterior myomas, a vertical incision using a unipolar hook is preferred (38). After an incision is made, the pseudocapsule and myoma can be visualized. A high voltage is recommended for the initial cutting, although studies have suggested a low voltage to preserve the myometrium (39). Moreover, studies have recommended certain incisions for easier suturing: sagittal for posterior nodules, oblique for anterior nodules, and transverse or elliptical to avoid excessive myometrial tissue (39, 40).

Conventional sutures include an atraumatic needle with a fine resorbable suture. Sutures can be performed in a single plane when the stitch can pass through the entire thickness (39). If the incision runs deep or the cavity has been opened, the suture will be performed in multiple layers. Surgeons can perform separate or continuous stitches, depending on a case-to-case basis. Intracorporeal or extracorporeal knots can be used if tension is maintained. Preformed disposable endoscopic loops can be used if a progressive tie can be realized (41). If enucleation is achieved, the endoloop is tied and offers mechanical hemostasis, as well as a good exposure of the cleavage plane. Studies have shown a reduction in diathermy, thus improving scar quality (41). The “bottom-up suture” technique was proposed for better suturing by elevating the bed of the myoma while still attached to the uterus (42); advantages of this technique include hemorrhage control and prevention of dead-space formation. The “baseball” suture technique has been described as an alternate option to the classical suture, affording advantages such as reduced suturing time, simple to perform, single-layer suture, reduced dead-space formation, and complete closure of the incision (43). In this technique, the needle is inserted initially into the bottom of the incision on each side leading to a final aspect similar to the stitches on a baseball.

Barbed sutures are among the most commonly used types of sutures. The major advantage is the ability to maintain tension by suturing and the lack of necessity for knots. This material is preferred for laparoscopic myomectomy, as multiple studies have shown the benefits of its use. Unidirectional barbed sutures with intracorporeal knots are associated with a shorter uterine wall repair time and significantly lower hemoglobin drop and blood loss than conventional sutures (44). One notable advantage is the ability to maintain the same tension of the uterine tissue during suturing, given the presence of barbs on the filament. These barbs create an equal distribution of force without the potential of tear- or laceration-induced damage around the knots. Collectively, these factors highlight a statistically significant reduction in surgical time (*p* < 0.0001) (40). Reduced technical difficulties related to suture characteristics and the learning curve have been reported (45, 46).

Nevertheless, laparoscopic blood loss remains a major challenge. Efficient hemostasis can be achieved by applying tension on the suture and suturing speed. Additional factors related to blood loss include the surgeon’s experience, fibroid incision, number and size of nodules, and previous medical treatment to reduce uterine bleeding (47). A meta-analysis (45) has revealed a significant reduction in blood loss during surgery in patients who received barbed sutures (*p* = 0.183). Although this type of suture is expensive, the cost benefits favor barbed sutures (48). One complication of barbed sutures is increased adhesion. The current data showed similar postoperative adhesions in six-month second-look laparoscopy (49). Overall, barbed sutures are advantageous and have been associated with reduced technical difficulty, enhanced safety, decreased closure time, and minimal blood loss (50). To improve this suture, a bidirectional knotless barbed suture has been proposed (51), with the same benefits mentioned previously.

Overall, studies suggest that the modified extracorporeal knot-tying technique and the use of barbed sutures may be associated with shorter operation times and less blood loss during laparoscopic myomectomy. However, further research is needed to confirm these findings and determine the best suture technique for individual patients.

## 3. The impact of suturing technique on uterine vascularity and scar repair

A correlation has been suggested between the suture technique and uterine scar healing. This correlation has been studied extensively regarding cesarean sections, owing to its association with uterine rupture.

The first step that can alter the healing process is the extensive application of bipolar coagulation, which leads to thermal tissue damage (52). It is recommended that electrosurgery must be limited as much as possible (53). In addition, excessive electrosurgery to control bleeding after suturing has been associated with the weakening of the suture material (54). The type of suture realized is related to scar healing and subsequent complications. Studies have evaluated the closing technique in cesarean sections, revealing that although single-layer closure has a shorter operative time (55), it can be associated with a higher risk of complications, such as scar defects (56). Compared with interrupted sutures, continuous sutures were associated with a high risk of uterine rupture (57) and placenta accreta (58).

Studies have compared different suturing techniques for laparoscopic myomectomy scar healing. Although the wound completely healed in approximately 3 to 6 months (59), the continuous suture scar failed to recover completely, accompanied by altered vascularity (60). Compared with interrupted sutures 3 months post-surgery, currently used continuous sutures in laparoscopic myomectomy were associated with excessive myometrial ischemia, delayed post-surgery vascularization, and persistent avascular areas at 6 months post-surgery (60).

Although uterine hemostasis is well achieved, long-term wound healing with continuous barbed sutures needs to be established when compared with that of interrupted sutures. Double-layered sutures are reportedly associated with fewer complications than single-layer sutures.

## 4. Impact of sutures type on fertility and pregnancy outcomes

Fertility preservation remains one of the main purposes of laparoscopic myomectomy. Reproductive outcomes must be considered when evaluating the advantages or disadvantages of the procedure. Uterine rupture and abnormal placentation are two of the most undesirable complications, and precise suturing is critical to avoid these complications (17, 45). The two main aspects of uterine repair are represented by the type of suture performed and the layers of suturing depending on the depth of the fibroid nodule.

A meta-analysis made by Gardella et al. evaluated the importance of barbed sutures on the fertility outcome and concluded that is not sufficient information regarding the long-term outcome (45). As the authors concluded, the available data regards patients with previous barbed suture, lacking information about fertility outcomes in control groups. Considering barbed suture outcomes, pregnancy rates of 71% have been reported (61), similar to other studies that evaluated laparoscopic myomectomy without differentiation (17, 62). Cesarean sections were performed frequently, and the reported complications included preterm birth, abnormal placentation, hypertensive disorders, growth restriction, and myoma degeneration (18 of 110 pregnancies) (61).

The degree of myometrial penetration during myomectomy has been correlated with scar formation but not with uterine rupture during pregnancy (63). Multiple-layer suturing of the uterine fibroid bed has been associated with better reproductive outcomes (64). Additionally, studies have reported similar outcomes between single- and multiple-layer suturing techniques (65). Abdominal myomectomy has been associated with higher cesarean section rates than laparoscopic myomectomy (42.1% vs. 89.5, *p* < 0.001) (66).

Minimally invasive procedures can treat most of the fibroids, but they can also lead to irreversible infertility (67). Pre-operative medical treatments can be used to minimize the specific complications and to optimize the results (68). Even though there are important risks related with this procedures, the presence of uterine leiomyomas during pregnancy influences the evolution and outcomes (69, 70). With or without surgery, uterine fibroids represent an risk for infertility that needs to be proper addressed (71).

## 5. Conclusion

Laparoscopic myomectomy can be a valuable strategy for patients who desire fertility preservation. With advantages such as minimal postoperative pain, rapid recovery, esthetic outcomes, and good reproductive outcomes, the laparoscopic approach is considered the leading surgical approach in this field. Nevertheless, there is still a need for large-scale multicenter studies comparing different surgical techniques and suture methods in females throughout pregnancy.

## Author contributions

MD, C-GN, and A-EN designed the study. MC, A-EN, and FȘ reviewed the literature and drafted the manuscript. MD, MC, AC, C-GN, and AN substantially contributed to the conception of the study and revised and edited the final manuscript. All authors have read and approved the final version of the manuscript.

## Conflict of interest

The authors declare that the research was conducted in the absence of any commercial or financial relationships that could be construed as a potential conflict of interest.

## Publisher’s note

All claims expressed in this article are solely those of the authors and do not necessarily represent those of their affiliated organizations, or those of the publisher, the editors and the reviewers. Any product that may be evaluated in this article, or claim that may be made by its manufacturer, is not guaranteed or endorsed by the publisher.
