# Corrigendum: Laparoscopic myomectomy – The importance of surgical techniques

**DOI:** 10.3389/fmed.2023.1251421

**Published:** 2023-08-21

**Authors:** Mihai Cristian Dumitraşcu, Cătălin-George Nenciu, Adina-Elena Nenciu, Amalia Călinoiu, Adrian Neacşu, Monica Cîrstoiu, Florica Şandru

**Affiliations:** ^1^Department of Obstetrics and Gynecology, “Carol Davila” University of Medicine and Pharmacy, University Emergency Hospital of Bucharest, Bucharest, Romania; ^2^Department of Obstetrics and Gynecology, University Emergency Hospital of Bucharest, Bucharest, Romania; ^3^Department of Obstetrics and Gynecology, “St. John” Emergency Clinical Hospital of Bucharest, Bucharest, Romania; ^4^Department of Internal Medicine, “Prof. Dr. Agripa Ionescu” Emergency Hospital, Bucharest, Romania; ^5^Department of Dermatology, Carol Davila' University of Medicine and Pharmacy, Bucharest, Romania; ^6^Department of Dermatology, Elias Emergency University Hospital, Bucharest, Romania

**Keywords:** laparoscopic myomectomy, suture, surgical technique, barbed suture, pregnancy outcome

In the published article, there was an error in the Acknowledgments statement. Support from the University of Medicine and Pharmacy Carol Davila was not mentioned. The correct Acknowledgments statement appears below.

The authors apologize for this error and state that this does not change the scientific conclusions of the article in any way. The original article has been updated.

